# BcGR1.1, a Cytoplasmic Localized Glutathione Reductase, Enhanced Tolerance to Copper Stress in *Arabidopsis thaliana*

**DOI:** 10.3390/antiox11020389

**Published:** 2022-02-15

**Authors:** Yan Li, Feiyi Huang, Yu Tao, Ying Zhou, Aimei Bai, Zhanghong Yu, Dong Xiao, Changwei Zhang, Tongkun Liu, Xilin Hou, Ying Li

**Affiliations:** State Key Laboratory of Crop Genetics & Germplasm Enhancement, Key Laboratory of Biology and Genetic Improvement of Horticultural Crops (East China), Ministry of Agriculture and Rural Affairs of the P. R. China, Engineering Research Center of Germplasm Enhancement and Utilization of Horticultural Crops, Ministry of Education of the P. R. China, Nanjing Agricultural University, Nanjing 210095, China; 2019204034@njau.edu.cn (Y.L.); hfy@njau.edu.cn (F.H.); 2020104058@stu.njau.edu.cn (Y.T.); 2018104059@njau.edu.cn (Y.Z.); 2021204026@njau.edu.cn (A.B.); 2018204024@njau.edu.cn (Z.Y.); dong.xiao@njau.edu.cn (D.X.); changweizh@njau.edu.cn (C.Z.); liutk@njau.edu.cn (T.L.); hxl@njau.edu.cn (X.H.)

**Keywords:** non-heading Chinese cabbage, *BcGR1.1*, GR, copper stress, ROS

## Abstract

Copper is a mineral element, which is necessary for the normal growth and development of plants, but high levels of copper will seriously damage plants. Studies have shown that *AtGR1* improves the tolerance of *Arabidopsis* to aluminum and cadmium stress. However, the role of *GR* in the copper stress response of plants is still unclear. Here, we identified four genes (named *BcGR1.1*, *BcGR1.2*, *BcGR2.1* and *BcGR2.2,* respectively) encoding glutathione reductase (GR) in non-heading Chinese cabbage (*Brassica campestris (syn. Brassica rapa) ssp. chinensis*), which could be divided into two types based on the subcellular localization. Among them, *BcGR1.1*, which belonged to the cytoplasmic localization type, was significantly upregulated under copper stress. Compared to WT (the wild type), *Arabidopsis thaliana* heterologously overexpressed *BcGR1.1* had longer roots, higher fresh weight, higher GSH levels and GSH/GSSG (oxidized form of GSH) ratio, and accumulated more superoxide dismutase and peroxidase under copper stress. However, in the AsA-GSH cycle under copper stress, the contents of AsA and AsA/DHA were significantly downregulated, and the contents of DHA and T-AsA (total AsA) were upregulated, in the *BcGR1.1*-overexpressing *Arabidopsis*. Therefore, *BcGR1.1* could improve the scavenging ability of reactive oxygen species (ROS) by increasing the activity of GR, antioxidant enzymes and the utilization of AsA, and then enhance the copper stress tolerance of plants.

## 1. Introduction

In addition to the common abiotic stresses in agricultural production, such as drought, flooding and extreme temperatures [1,2], heavy-metal pressure has become another stress threatening crop production [3]. This is mainly due to the unrestricted industrialization and urbanization that has occurred in the last few decades. Heavy-metal pollution has become the focus of global attention due to its wide range, strong toxicity, long-term effects and irreversibility [4,5]. The heavy metals with the greatest influence on plant metabolism are Pb, Cd, Cu and Zn. As an essential heavy-metal element [6], copper plays a key role in many biological processes of plants, including photosynthesis and respiratory electron transfer, cell wall remodeling, superoxide scavenging, lignification and ethylene sensing [7,8,9,10,11,12,13]. If the copper content in plants is insufficient, the growth and development of plant reproductive organs are affected [14]. However, when the concentration of copper exceeds the requirements for plant growth, the structure and function of the plant cell membrane will be damaged, the permeability of plant cell membranes will be affected, and the antioxidant enzyme system and chloroplast structure of plants will be damaged, resulting in inhibition of the growth and development of plants [15,16,17,18,19,20,21].

Under copper stress conditions, the growth of plant primary roots is inhibited [22], which, in turn, affects the growth and development of aerial parts. Under the conditions of copper stress, there will be an excessive accumulation of ROS in plants, which is toxic to plants [23]. In the process of plant evolution, some mechanisms for coping with copper stress have been developed, including adjusting the dynamic balance of copper [24], activating the antioxidant defense response [25], and so on. The defense system includes enzymes that remove ROS, such as superoxide dismutase (SOD), peroxidase (POD), and catalase (CAT) as well as low-molecular-weight antioxidants, such as reduced ascorbic acid (AsA) and reduced glutathione (GSH) [26].

As an important component of the antioxidant system [27], GSH can play an important role in the physiological activity of organisms. It actively participates in the formation of disulfide, sulfide, thiolipid and other sulfides. It also participates in the elimination of excess ROS [28], the reduction in peroxides, the transmission of redox-sensitive signals [29] and the elimination of superoxide free radicals in the process of cell metabolism. It can coordinate with heterologous toxic substances, regulate plant growth and development, and resist various stresses (e.g., temperature, heavy metals, osmotic stress, and pathogen infection) [30,31,32,33,34,35,36,37,38]. GSH can chelate with copper in plants to excrete copper from the plant, thereby significantly reducing the copper content in plant tissues [39]. In addition, GSH is the electron donor of oxidized ascorbic acid (DHA), and its redox state is associated with the AsA-GSH cycle. As the main antioxidant system in plants, the AsA-GSH cycle can inhibit the production of ROS. This plays an important role in plant anti-aging and stress adaptation [40].

In general, glutathione mainly exists in two completely different forms: the reduced form (GSH) and the oxidized form (GSSG). Glutathione in plants is mostly reduced [41]. Therefore, glutathione is commonly referred to as the reduced form (GSH). In the face of stress, the activity of GR in an organism is improved, and the content of GSH is increased by the catalytic reduction in GSSG to increase plants’ ability to resist environmental stress [42,43,44,45]. Therefore, high GR activity is necessary for plants to maintain high levels of GSH, especially under stress conditions.

In recent years, arable land area is decreasing. Thus, how to safely use the heavy-metal-contaminated arable land has become a major problem. However, since plants are immovable, they lack the ability to avoid the polluted environment. Therefore, the only chance for them to survive under adverse conditions is the mobilization of defense mechanisms and the evolution of a more tolerant genotype [46]. In *Arabidopsis thaliana*, overexpression of *AtGR1* results in high levels of GSH and GSH/GSSG ratios that help suppress ROS and lipid-peroxide-derived reactive carbonyl species (RCS) damage to plants, and enhance the dual detoxification function of plants, thereby enhancing the aluminum tolerance of *Arabidopsis thaliana* [47]. However, unlike aluminum stress, copper, as one of the components of heavy-metal pollution, is itself an essential trace element for plants. Therefore, it is particularly important to improve the plants’ ability to cope with copper stress. The copper content in the soil should be not too much or too little. Therefore, it is particularly important to study the coordinated evolution of copper in plants and soils.

In this study, we identified the location of *BcGR*s in non-heading Chinese cabbage by subcellular localization. The expression patterns of *BcGR*s under copper stress were analyzed, of which, *BcGR1.1* was significantly upregulated. To determine whether *BcGR1.1* could affect the tolerance of plants to copper stress, we silenced *BcGR1.1* in non-heading Chinese cabbage and overexpressed it in *Arabidopsis*. Our results showed that the overexpression of *BcGR1.1* led to a high concentration of GSH and a high ratio of GSH/GSSG in tissues. This improved AsA utilization and the activities of antioxidant enzymes, which contributed to the effective removal of ROS in plants, and improved plant tolerance to copper stress. This study was of great significance for the cultivation of new varieties of copper-stress-tolerant crops, and made the utilization of heavy metal contaminated soil possible.

## 2. Materials and Methods

### 2.1. Phylogenetic and Structural Analysis of GRs

The protein sequences of *BcGR*s were translated by BioXM2.7 according to the cloned CDS sequences. The protein sequences of GR in *Brassica rapa*, *Brassica oleracea var. Oleracea*, *Eutrema salsugineum*, *Raphanus sativus*, *Arabidopsis thaliana*, *Capsella rubella*, *Camelina sativa* and *Brassica napus* were downloaded from NCBI (https://www.ncbi.nlm.nih.gov/. accessed on 21 November 2021). A conserved structure analysis performed by MEME and MEGA 7.0 was used for evolution analysis.

### 2.2. Plant Materials and Growth Conditions

The seedlings of non-heading Chinese cabbage cultivar (*Brassica rapa ssp. Chinensis* cv. Suzhouqing) used in this study were grown in an artificial chamber (Ningbo Southeast Instrument Co., Ltd. Ningbo, China) with a 16-h/8-h light/dark cycle at 24 °C/18 °C ± 1 °C, 65% humidity ±7%. Non-heading Chinese cabbage seeds were first sown in a 32-hole tray; one-month-old seedlings were grown in Hoagland nutrient solution for copper treatment or transplanted into bigger square flowerpot for virus-induced gene silencing (VIGS).

*Arabidopsis thaliana* Col-0 plants were grown in a long-day artificial chamber (Ningbo Southeast Instrument Co., Ltd. Ningbo, China) with a 16-h/8-h light/dark cycle at 22 °C/18 °C ± 1 °C, and 75% humidity ±7%. Arabidopsis seeds were evenly spread on 1/2 MS for growth, after disinfection. The seed disinfection process was: 75% (*v*/*v*) alcohol cleaning for 1 min, followed by sterile water cleaning 3–5 times, 5 min each time. Then, the seeds were washed with 10% (*v*/*v*) sodium hypochlorite for 10 min, followed by 3–5 washes with sterile water for 5 min each. The whole process was completed in the clean bench.

Tobacco seeds were first sown in a square flowerpot and placed in a 24 °C/19 °C, 70% humidity and 16 h/8 h light/dark cycle climate chamber for two weeks. The seedlings were then transplanted into a 32-hole tray for the transient transformation test.

### 2.3. Copper-Stress Treatment and Sampling

For copper-stress treatment of non-heading Chinese cabbage, one-month-old seedlings were cultured in Hoagland nutrient solution containing 100 µM CuSO_4_·5H_2_O for 48 h. The leaves were harvested as samples at 0, 3, 6, 9, 12, 24, 36, 48 h (0 h was used as a control).

For the copper-stress treatment of *Arabidopsis thaliana*, we used WT plants to carry out the pre-experiment. Three-day-old wild-type seedlings were transferred onto 1/2 MS medium containing 0, 25, 50, 75, 100 µM CuSO_4_·5H_2_O, respectively. The pictures were taken 15 d after treatment (Figure S2). According to our pre-experiment results, 75 µM was selected as the optimal treatment concentration. Three T3 generation *BcGR1.1*-OE transgenic lines and the wild-type Arabidopsis seeds were sterilized and seeded on 1/2 MS medium. The seeds were incubated in dark at 4 °C for 2 days to break dormancy. After 3 days, the seedlings were transferred to 1/2 MS medium with or without 75 µM CuSO_4_·5H_2_O. After 21 days of cultivation, the root length and fresh weight (FW) of the plants were measured.

To further determine the tolerance to excess copper in *BcGR1.1*-OE transgenic plants, after two weeks of normal culture, plants with uniform growth were selected and transferred into 1/2 MS medium with or without 75 µM CuSO_4_·5H_2_O for 24 h. Samples were collected to determine GR, GSH, GSSG, AsA and antioxidant enzymes.

### 2.4. Subcellular Localization Analysis

The coding region of the *BcGR*s was introduced into the pRI101-GFP vector digested by *Bam*HI and *Nde*I restriction endonucleases, and the construct 35S: *BcGR*-GFP was obtained. The recombinant plasmid and empty vector were transformed into *Agrobacterium* GV3101; individual Agrobacterium colonies were grown for 20 h in 500 µL cultures (Luria broth, 50 µg/mL rifampicin, 50 µg/mL kanamycin) at 28 °C. After identification of PCR, they were inoculated in 5 mL culture (Luria broth, 50 µg/mL rifampicin, 50 µg/mL kanamycin) and grown at 28 °C for 16–20 h. Bacteria were pelleted by centrifugation, resuspended in infiltration medium (10 mM MgCl_2_, 10 mM Mes, 150 µM acetosyringone, pH 5.7) to OD600 = 0.8, and incubated at room temperature for at least 4 h. Using a 1 mL syringe, the Agrobacterium solution was injected from the back of the tobacco leaf into the tobacco by pressure osmosis.

After injection of Agrobacterium, normal culture for 48–60 h. *N. benthamiana* cellular images were then taken by a confocal laser-scanning microscope (Zeiss, lsm780, Jena, Germany). GFP and chloroplast autofluorescence are excited by 488 nm excitation light, the fluorescence collection wavelengths are 500~530 nm (GFP) and 650~750 nm (chloroplast fluorescence), respectively, the RFP excitation light is 543 nm, and the fluorescence collection wavelengths are 560~630 nm.

### 2.5. PCR and qRT-PCR Analysis

We cloned *BcGR*s by PCR using cDNA of non-heading Chinese cabbage as the template, and the DNA of non-heading Chinese cabbage was used as the template for the cloning of pro*BcGR*s. Gene-cloning-specific primers were designed based on the CDS sequence of *GR* from the non-heading Chinese cabbage database (http://nhccbase.njau.edu.cn/website/ accessed on 21 November 2021) and the chromosome-level reference genome of non-heading Chinese cabbage [48]. *BcGR1.1*-OE transgenic Arabidopsis was also identified by PCR. PCR amplification conditions were the same, 94 °C, 5 min, 94 °C, 30 s, 58 °C, 30 s, 72 °C, 2 min, 72 °C, 10 min, and incubation at 4 °C. Steps 2–4, 35 cycles.

The total RNA of non-heading Chinese cabbage and *Arabidopsis thaliana* were extracted using the Total RNA Extraction Kit (Tiangen, Beijing, China). The RNA was reverse transcribed into cDNA by a PrimeScript™RT reagent Kit with gDNA Eraser (Takara, Dalian, China). qRT-PCR was performed using the SYBR Premix ExTaq kit^®^ (TaKaRa) according to the manufacturer’s instructions. The instrument used for real-time quantification was the ABI StepOnePlus™ Real-Time PCR System (Applied Biosystems, Foster City, CA, USA). PCR reaction mix contained 10 µL of 2×SYBR ^®^ Premix Ex Taq TM II (TaKaRa), 6.8 µL of ddH_2_O, 0.4 µL of ROX Reference Dye II, 0.8 µL of each gene-specific primer and 2.0 µL of diluted cDNA. The PCR procedure was as follows: predenaturation, 95 °C 30 s, 1 cycle. 2 step PCR: 95 °C 5 s, 60 °C 30 s, 40 cycles. In addition, the melting curve was used to verify the specificity of all reactions. *BcGAPC* and *AtActin* were used as the internal standard, respectively; all primers used for qRT-PCR are listed in Table S1. The 2^−∆∆Ct^ method was used for data calculation [49]. The experiments were repeated three times independently for biological replication.

### 2.6. VIGS in Non-Heading Chinese Cabbage

VIGS was carried out to obtain *BcGR1.1*-silencing plants according to the previous report [50]. The 80 nt specific palindrome sequence (5′-CTGACGGAGAGCTTGACAAGGCGGTGGCGGCTGAGGAAGCGCTTCCTCAGCCGCCACCGCCTTGTCAAGCTCTCCGTCAG-3′) was designed based on the *BcGR1.1* coding sequence and sent to the genescript company (Nanjing, China) for synthesis. The PTY vector was digested by the restriction endonuclease *Sna*BI. The linearized PTY vector was ligated with the synthesized 80 nt specific palindrome sequence by T4 ligase, and then transformed into *E. coli* DH5α. The correct recombinant plasmid was obtained by ampicillin LB plate screening and bacterial liquid PCR detection and sequencing. A high number of plasmids were extracted. Then, 50 µg of PTY and PTY-*BcGR1.1* plasmids were wrapped in gold particles, respectively, and bombarded into two-week-old seedlings of non-heading Chinese cabbage using gene-gun-mediated transformation (1300 psi, PDS-1000/He, Bio-Rad, Hercules, CA, USA). Then, the non-heading Chinese cabbage plants bombarded by the gene gun were grown in an artificial chamber (Ningbo Southeast Instrument Co., Ltd. Ningbo, China) with a 16-h/8-h light/dark cycle at 24 °C/18 °C (temperature fluctuation: ±1 °C), 65% humidity (humidity fluctuation: ±7%) for 15–20 days. Virus-infected seedlings exhibited mosaic leaves, then samples were taken for qRT-PCR. The primers used are listed in Table S1.

### 2.7. Agrobacterium-Mediated Transformation of Arabidopsis

Transgenic Arabidopsis plants were obtained by *Agrobacterium*-mediated floral dip transformation according to the methods reported by Clough and Bent [51]. The CDS of *BcGR1.1* was inserted into the PTCK303 vector and the recombinant plasmid was then transformed into *Agrobacterium* GV3101. The harvested T0 generation seeds were screened on an MS medium containing hygromycin and temetine. The positive T1 lines were further confirmed by PCR, GUS (β-d-glucuronidase) staining and real-time quantitative PCR. The subsequent T2/T3 generations were screened and identified according to the same methods. Finally, the T3 generation displaying stable inheritance was used as the experimental material to study the function of *BcGR1.1*.

To analyze the tissue expression difference of *BcGR1.1*, we cloned the promoter of *BcGR1.1*, recombined the promoter sequence with pCAMBIA1301 vector digested by *Pst*I and *Nco*I, and then the positive plants were used for GUS analysis.

### 2.8. Determination of Phenotypic and Physiological Indexes

Phenotypic indices included fresh weight and root length. For the fresh weight of whole-plant, 10 *Arabidopsis thaliana* plants were taken from the treatment and control groups, respectively. Root length is measured from the base of the root to the longest position of the root. When measuring, we first took the treated seedlings from the medium, spread the root system on a piece of white paper, recorded the position of the root base and root tip, drew a straight line, and measured the root length with a ruler.

Physiological indexes include the contents of GSH, GSSG, AsA, DHA, H_2_O_2_ and the activities of antioxidant enzymes (SOD, POD, CAT) and GR. GR [52], GSH [53], GSSG [53], POD [54] and H_2_O_2_ [55] were determined with kits (Solarbio, Beijing, China). The Superoxide Dismutase Detection Kit (A001. Nanjing Jiancheng Bioengineering Institute, Nanjing, China) and The Catalase Detection Kit (A007. Nanjing Jiancheng Bioengineering Institute, Nanjing, China) were selected for SOD and CAT measurement. These assays were conducted according to the manufacturer’s instructions. The AsA content in the leaves was determined according to the method reported by Fontannaz et al. [56]: taking 0.2 g leaves, grinding them into powder, adding 1.5 mL of 0.1% oxalic acid, mixing well, 4 °C, 12,000 r·min^−1^, centrifugation for 20 min, and filtering the centrifuged supernatant with a 0.22 µm water filter. After diluting twice with 0.1% oxalic acid, the filtered supernatant was taken using an ultra-high-performance liquid chromatograph (UltiMate 3000) to determine the content of AsA. Chromatographic conditions: mobile phase 0.1% acetic acid, flow rate 1 mL/min, injection 10 µL, column temperature 30 °C, detection wavelength 245 nm. After ASA detection was complete, an equal volume of 20 mg/mL DTT was added, and tested again to obtain T-AsA. A standard curve was made with different concentrations of AsA, and a regression equation was obtained to convert the actual AsA concentration of the samples. DHA content is the difference between T-AsA and AsA content. During the whole AsA extraction process, attention should be paid to low temperatures and light should be avoided to prevent degradation.

### 2.9. Statistical Analysis

Each experiment was carried out with three or four independent biological replicates. Data were subjected to ANOVA using IBM SPSS Statistics 21 software. Before performing an ANOVA, we studied the normality and homoscedasticity of the data. Differences between mean values were compared by LSD test (*p* < 0.05).

## 3. Results

### 3.1. Phylogeny and Conserved Domains Analysis of GRs

**Figure 1:**
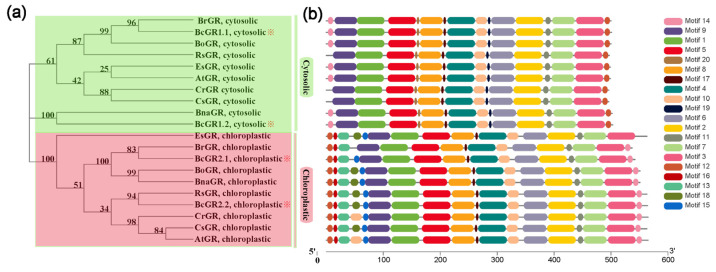
Phylogenetic relationships (**a**) and conserved motif distributions (**b**) of GRs. Each motif is represented by a coloured box numbered at the right.

Four *GR* genes in non-heading Chinese cabbage were identified, and named *BcGR1.1*, *BcGR1.2*, *BcGR2.1* and *BcGR2.2*. The results of phylogenetic tree analysis and the differential distribution of conserved motifs indicate that the GRs of non-heading Chinese cabbage are similar to other varieties, with two subcellular localization types: cytoplasm (Figure 1a, green box) and chloroplast (Figure 1a, pink box). Phylogenetic tree analysis showed that there is a close evolutionary relationship among non-heading Chinese cabbage, *Brassica rapa*, *Brassica napus* and *Raphanus sativus* (Figure 1a). Motif analysis showed that the motifs of *BcGR*s are overwhelmingly the same as those of other species (Figure 1b). For the cytoplasmic localized GR, there was only a difference in the first motif, motif 13, which was only identified in several other species, except RsGR, CrGR, and CsGR. Chloroplast-localized GR, according to the difference between the fourth and fifth conserved motifs, can be divided into four categories. EsGR, BoGR, BnaGR, RsGR, BcGR2.2, CsGR have exactly the same motifs and belong to the same class; compared with this class, BrGR lacks motif 18 and motif 15, and BcGR2.1 does not contain motif 18, The fourth motif of CrGR and AtGR is motif 10 instead of motif 18.

### 3.2. Subcellular Localization of BcGRs

**Figure 2:**
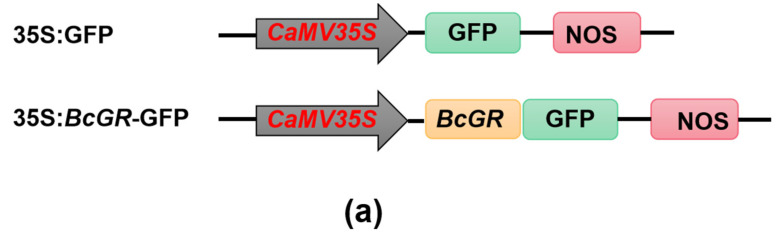

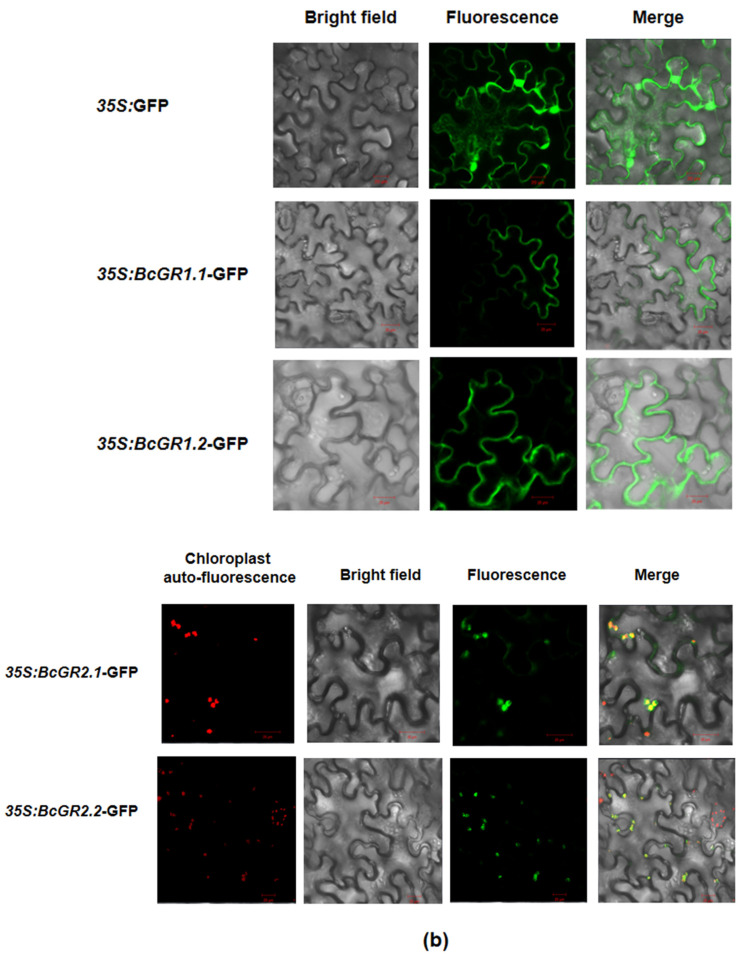
Subcellular localization of *BcGR*s in *Nicotiana benthamiana* plants. (**a**) Schematic diagram of the construction of subcellular localization vector. (**b**) *N. benthamiana* cellullar images of *BcGR*s. We used 35S:GFP empty vector as a control in this experiment, and fused *BcGR*s protein with GFP to observe their subcellular localization. Chloroplast autofluorescence, red fluorescence of chloroplasts under excitation light at 488 nm and collection light at 650–750 nm. Bright field, bright field images of tobacco leaf cells. Fluorescence, green fluorescence of fusion protein of 35S: BcGR-GFP or 35S: GFP. Merged, overlay of bright field, green fluorescence, or bright field, green fluorescence and red fluorescence images. Bars = 20 µm.

To further determine the specific location of BcGR proteins in cells, subcellular localization was performed. We constructed a vector (Figure 2a), fused *BcGR*s with GFP, and detected the localization of *BcGR*s protein in cells by observing GFP. Results show that GFP protein was expressed in the whole cell, while BcGR1.1-GFP and BcGR1.2-GFP fusion proteins were mainly expressed in the cell membrane and cytoplasm, BcGR2.1-GFP and BcGR2.2-GFP fusion proteins were mainly expressed in the chloroplast (Figure 2b). This result indicated that the non-heading Chinese cabbage had two types of GR proteins: cytosolic localization and chloroplastic localization, which is consistent with the results of evolutionary analysis.

### 3.3. Expression Patterns of BcGRs

**Figure 3:**
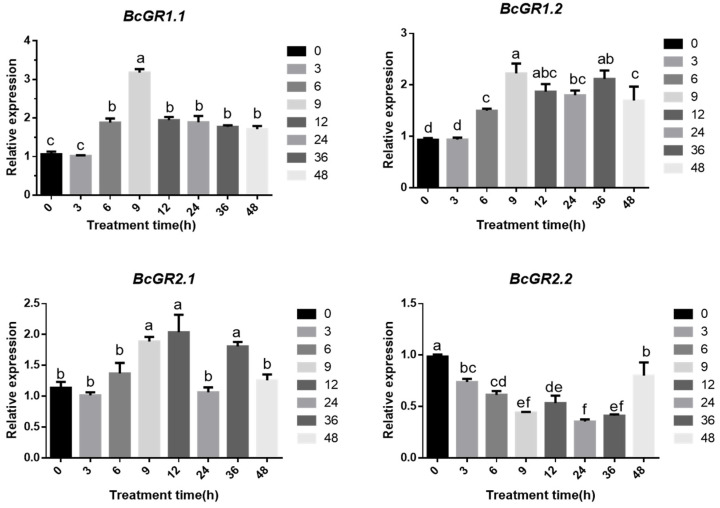
Expression patterns of *BcGR*s genes in the non-heading Chinese cabbage under copper stress. Analysis of *BcGR*s expression at different time points (0 h, 3 h, 6 h, 9 h, 12 h, 24 h, 36 h and 48 h) under copper stress. The expression levels of *BcGR*s represented the expression fold of each *BcGR* in seedlings treated with copper stress at different times, relative to seedlings without copper stress treatment, using the *BcGAPC* gene as an internal reference gene. The expression level of *BcGR*s at each timepoint is the average of three replicates. Error bars indicate standard error of mean (SEM) of three independent experiments. According to the LSD test, the values within the same treatment followed by the same letter are not significantly different (*p* < 0.05).

Here, we used one-month-old, non-heading Chinese cabbage for copper treatment. The qRT-PCR showed that the four *GR* homologous genes in non-heading Chinese cabbage have different responses under copper stress (Figure 3). *BcGR1.1*, *BcGR1.2* and *BcGR2.1* were upregulated, especially *BcGR1.1*. The expression rapidly increased 6 h after copper stress, reaching a peak at 9 h and then slowly decreasing. However, the expression pattern of *BcGR2.2* was completely different from the other three *BcGR*s. *BcGR2.2* was downregulated, and showed the lowest expression at 9 h. The results showed that, among the four *GR* homologous genes, *BcGR1.1* may play a major role in copper stress.

### 3.4. Heterologous Overexpression of BcGR1.1 in Arabidopsis thaliana

**Figure 4:**
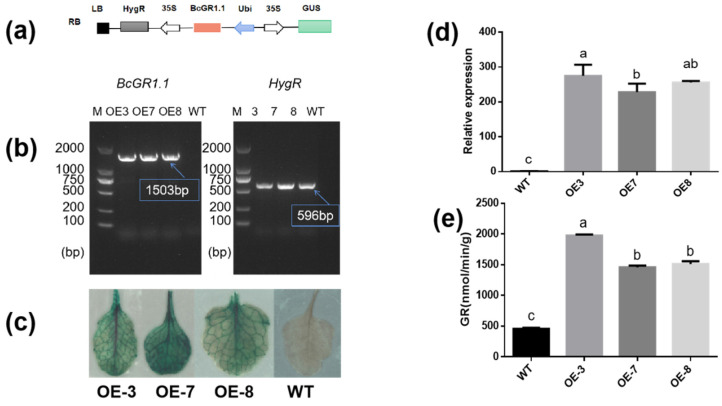
Creation and identification of *Arabidopsis thaliana* overexpressing *BcGR1* gene. (**a**) Construction of a vector expressing *BcGR1.1* under the control of Ubi promoter. (**b**) DNA level identification of positive seedlings. DNA from overexpression plants (OE3, OE7, OE8) and wild-type (WT) were used as PCR templates. Specific primers for *BcGR1.1* and hygromycin genes were used, and M represents DNA marker. (**c**) GUS detection of *BcGR1.1* in overexpression plants and WT. (**d**) Analysis of *BcGR1.1* expression level in overexpression plants and WT. (**e**) Detection of GR activity in overexpression plants and WT. Error bars indicate standard error of mean (SEM) of three independent experiments. According to the LSD test, the values within the same treatment followed by the same letter are not significantly different (*p* < 0.05).

To further study the function of *BcGR1.1*, we transformed *Arabidopsis thaliana* by dipping flowers to obtain *BcGR1.1*-overexpressing plants. For each generation of transgenic plants, DNA level detection (Figure 4b), GUS detection (Figure 4c) and expression analysis (Figure 4d) were performed, respectively. After obtaining the transgenic lines, we tested the GR activity in the WT and transgenic lines. The results showed that the expression levels of *BcGR1.1*-0E3, *BcGR1.1*-0E7, and *BcGR1.1*-0E8 were increased to 274, 228, and 255 times of the WT, and the GR activity were significantly higher than that of the WT, which were 4.3, 3.1, and 3.3 times that of the WT, respectively (Figure 4e).

### 3.5. Overexpression of BcGR1.1 Improved Copper Stress Tolerance in Arabidopsis thaliana

**Figure 5:**
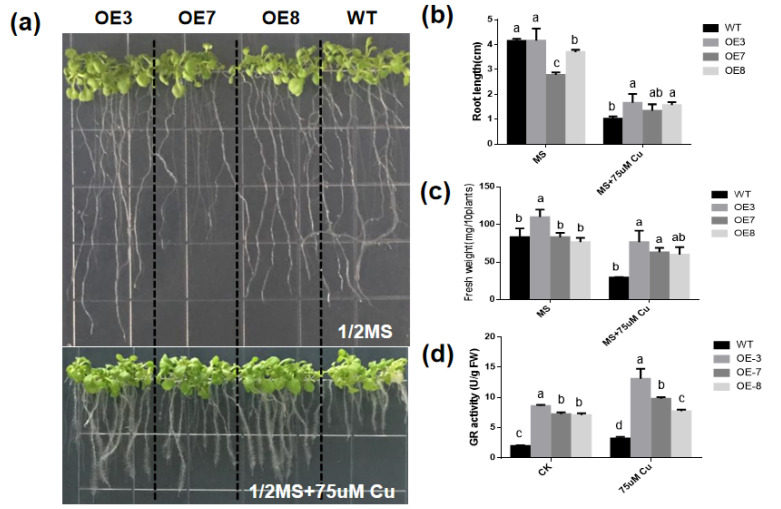
The growth state of *Arabidopsis thaliana* overexpressing *BcGR1.1* gene under copper stress. (**a**) Image of 21-day growth on half-strength Murashige and Skoog (1/2 MS) medium with or without 75 µM copper sulfate pentahydrate (CuSO_4_·5H_2_O). (**b**) Root growth. (**c**) Fresh weight. The fresh weight (FW) of wild-type and transgenic plants treated with or without 75 µM CuSO_4_·5H_2_O for 21 days. (**d**) Detection of GR activity in overexpression plants and WT. According to the LSD test, the values within the same treatment followed by the same letter were not significantly different (*p* < 0.05). Error bars indicate standard error of mean (SEM) of three independent experiments.

The expression of *BcGR1.1* was significantly upregulated under copper stress, and its heterologous overexpression in *Arabidopsis* obviously increased the GR activity. Therefore, we further studied the role of *BcGR1.1* in the response of plant to copper-stress. The GR, root length and fresh weight of WT and *BcGR1.1*-OE plants were measured under normal conditions (CK) and copper stress. Regardless of the presence of copper stress, the activity of GR in *BcGR1.1*-OE plants was always higher than that of WT. The GR activities of *BcGR1.1*-OE and WT were both increased under copper stress (Figure 5d). Under normal growth conditions, the roots of WT were longer than that of transgenic lines. Under copper treatment, root growth of WT and transgenic plant lines was significantly inhibited, and the root growth of WT plants was shorter than that of *BcGR1.1*-OE plants (the inhibition rates were 75% and 56%, respectively) (Figure 5b). There was no significant difference between the WT and *BcGR1.1*-OE plants under normal growth conditions, except OE3. However, the fresh weight of the transgenic lines was significantly higher than that of WT under copper treatment (Figure 5c). The results showed that the *BcGR1.1*-OE plants grew better under copper stress due to the longer roots and increased biomass.

### 3.6. Effects of BcGR1.1 Overexpression on the Status and Content of glutathione and AsA in A. thaliana

**Figure 6:**
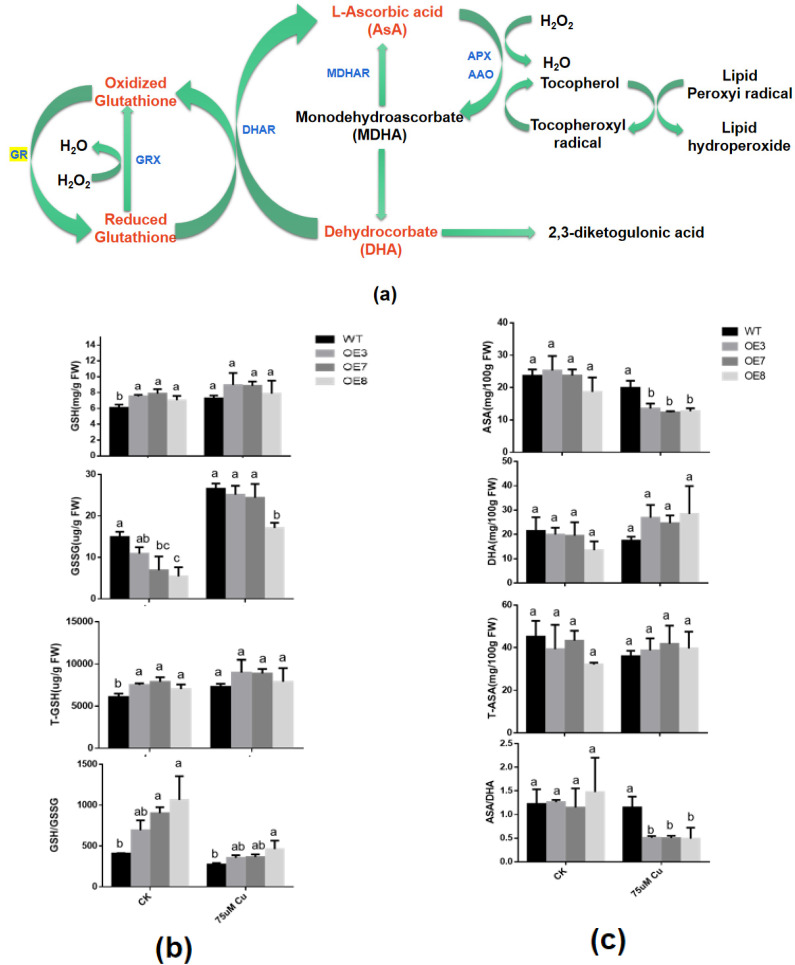
Analysis of antioxidant (AsA, GSH) content in WT and transgenic lines under copper stress. (**a**) The cycle of ASA-GSH. (1. ascorbate peroxidase (APX). 2. ascorbate oxidase (AAO). 3. monodehydroascorbate reductase (MDHAR). 4. dehydroascorbate reductase (DHAR). 5. glutathione peroxidase (GRX). 6. glutathione reductase (GR). (**b**) GSH, GSSG, T-GSH, and GSH/GSSG. (**c**) AsA, DHA, T-AsA and AsA/DHA. The plants were sampled and analyzed after exposure to normal or excess copper for 24 h. The data error is expressed as standard error of mean (SEM). According to the LSD test, there was no significant difference in the values with the same letter after the same treatment (*p* < 0.05).

In the cycle of ASA-GSH (Figure 6a) [57], ASA and GSH are both important reducing substances in plants. Their oxidation/reduction status is closely related to the stress tolerance ability of plants. Therefore, we analyzed the changes in the status and content of these two antioxidant substances under copper stress.

As far as glutathione is concerned, the GR enzyme activity in transgenic lines are significantly increased under normal growth conditions and under copper treatment (Figure 5d), which may result in a higher GSH content and lower GSSG and total glutathione (T-GSH) content. However, this difference is significant under normal growth conditions, but not under copper stress. This may be the result of the significant upregulation of *GR* in WT under copper stress. GSH/GSSG in both wild-type and transgenic plants decreased under copper stress. However, in transgenic lines, GSH/GSSG is still higher than WT (Figure 6b). These results indicated that higher levels of GSH may be beneficial for plants to cope with copper stress.

As for AsA, there is no significant difference in AsA, DHA, T-AsA and AsA/DHA content in WT and transgenic lines under normal conditions. Under copper stress, the AsA content in the transgenic line was significantly lower than that of the WT, while the content of DHA was the opposite. However, there was no significant difference between T-AsA. This resulted in a significant reduction in AsA/DHA in transgenic lines, to about 50% of the WT plants (Figure 6c). These results suggest that the heterologous overexpression of *BcGR1.1* in *Arabidopsis* may improve the efficiency of transforming AsA into DHA, thereby efficiently clearing ROS and improving plant tolerance to copper stress.

### 3.7. Antioxidant Enzyme Activities Are Altered in Transgenic A. thaliana

**Figure 7:**
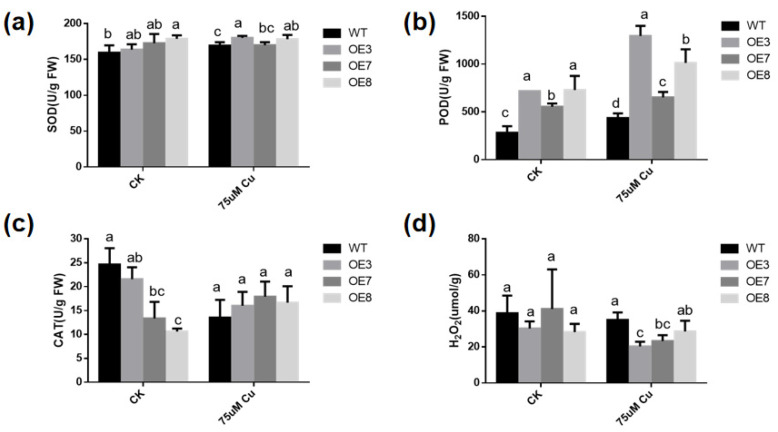
Determination of antioxidant enzyme activity. Analysis of the enzyme activities of SOD (**a**), POD (**b**), CAT (**c**) and the content of H_2_O_2_ (**d**) with or without excessive copper treatment. The plants were sampled and analyzed after exposure to normal or excess copper for 24 h. And the data error is expressed as standard error of mean (SEM). According to the LSD test, there was no significant difference in the values with the same letter after the same treatment (*p* < 0.05).

To determine whether the increase in copper-stress tolerance of transgenic plants is related to the change in antioxidant activity, the activities of SOD (Figure 7a), POD (Figure 7b), CAT (Figure 7c) and the content of H_2_O_2_ were measured in WT and transgenic plants with or without excessive copper treatment. With or without copper stress, the SOD and POD activities in *BcGR1.1*-OE plants were higher than those in WT. Under copper-stress treatment, SOD activity did not significantly change in WT and *BcGR1.1*-OE lines, but POD enzyme activity significantly increased. Under copper stress, the POD activities of OE3 and OE8 were 2.9 times and 2.3 times higher than that of WT, respectively. The CAT activity of *BcGR1.1*-OE line was lower than that of WT plants under normal conditions, but showed no difference under copper stress treatment. Upregulation of POD activity levels in transgenic lines under copper stress resulted in significantly lower H_2_O_2_ content in transgenic lines than in WT (Figure 7d). The results indicated that the heterologous overexpression of *BcGR1.1* in *Arabidopsis* can regulate the activity of certain antioxidant enzymes, reduce the ROS damage to plants, and improve the tolerance of plants to copper stress.

### 3.8. Virus-Induced BcGR1.1 Silencing in Non-Heading Chinese Cabbage

**Figure 8:**
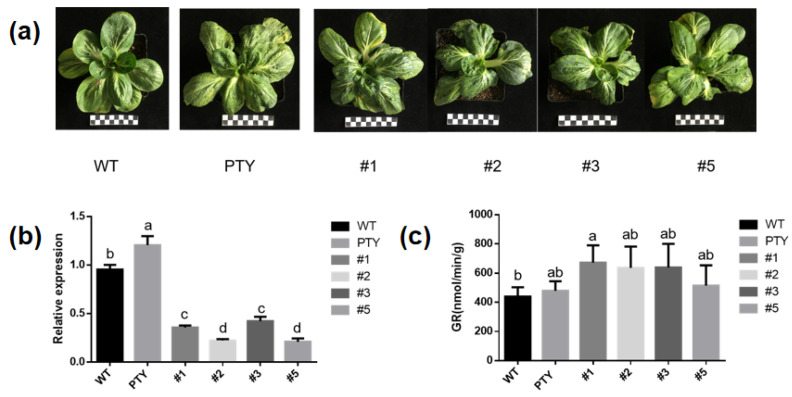
Virus-induced *BcGR1.1* silencing in non-heading Chinese cabbage. (**a**) Virus symptoms of the plants. (**b**) qPCR detection in *BcGR1.1* silenced plants. (**c**) Determination of GR activity of *BcGR1.1*-silenced plants. The data error is expressed as standard error of mean (SEM). According to the LSD test, the values within the same treatment followed by the same letter are not significantly different (*p* < 0.05).

We used VIGS experiment to further study the function of *BcGR1.1* in non-heading Chinese cabbage. Two weeks after virus inoculation, PTY-*BcGR1.1* and PTY showed a mosaic leaf phenotype (Figure 8a). Plants with potential *BcGR1.1* function loss were sampled, and qRT-PCR was used to evaluate the silencing efficiency of *BcGR1.1*. As shown in Figure 8b, compared with PTY plants, *BcGR1.1* expression was significantly reduced by 70.4%, 81.4%, 64.9% and 82.6% in pTY-*BcGR1.1*#1, #2, #3, and #5 seedlings, respectively. There was no significant difference in GR activity between PTY-*BcGR1.1* and PTY plants (Figure 8c), which may be the result of functional complementarity between *BcGR* homologous genes (Figure S1).

## 4. Discussion

In recent years, soil pollution caused by heavy metals has become a threat to agricultural production [58,59,60]. Excess heavy metals absorbed by the plant will threaten the normal metabolism of plants, hinder plant growth, and eventually lead to the decline in economic output. In addition, the accumulation of metal residues in the major food chains has been shown to cause serious ecological, environmental and health problems [61,62]. With the development of modern molecular biology, transgenic technology has become an effective means of finding new heavy-metal-tolerant genes in plants. There have been many studies on this; in Arabidopsis, *SIZ1* negatively regulates plant aluminum tolerance by mediating the STOP1-ALMT1 pathway [63]. The ectopic expression of *OsMYB*-R1 in Arabidopsis enhances tolerance to chromium stress [64]. In wheat, *WRKY74* affected GSH accumulation under Cu stress by regulating *GST1* expression, contributing to the amelioration of Cu toxicity [65]. There are also some studies on GR and heavy-metal detoxification. Endogenous salicylic acid improves the cadmium tolerance of Arabidopsis thaliana by regulating glutathione metabolism [66]. The overexpression of *AtGR1* enhances aluminum tolerance in Arabidopsis [47]. However, little is known about the relationship between GR and plant copper tolerance. Here, we identified the function of *BcGR1.1* under copper stress according to its heterologous expression in Arabidopsis.

GR is the only enzyme that catalyzes the reduction of GSSG to GSH. The Arabidopsis genome encodes two types of GR proteins: GR1 and GR2 [67]. The genome of non-heading Chinese cabbage also encodes two types of GR proteins, which have different subcellular locations (Figure 2). Studies have shown that cytoplasmic GR plays a key role in maintaining the redox state of the GSH pool after stress, rather than chloroplast localization GR [68]. Our results show that the expression of *BcGR1* is higher than that of *BcGR2* under copper stress, especially *BcGR1.1*(Figure 3). In addition, we found three similar sequences of metal response elements (MRE-like sequence: 5′-TGCAG-3′) [69] in the *BcGR1.1* promoter. This further indicated that *BcGR1.1* is involved in the process of non-heading Chinese cabbage against copper stress.

Based on the special performance of *BcGR1.1* under copper stress, we carried out in-depth research. However, due to the special genotype of non-heading Chinese cabbage, its tissue regeneration ability is very poor, and it is low efficiency to obtain the transgenic non-heading Chinese cabbage [70]. Therefore, we chose to overexpress *BcGR1.1* in Arabidopsis and silenced *BcGR1.1* in non-heading Chinese cabbage to determine whether *BcGR1.1* expression affects plant tolerance to copper. We detected that the expression level of *BcGR1.1* in transgenic *Arabidopsis* was about 252 times higher than that in WT plants, and the activity of GR was significantly higher than that of the WT (Figure 5d). This indicated that *BcGR1.1* can still function normally after heterologous overexpression in Arabidopsis. Therefore, we chose *BcGR1.1* overexpressing *Arabidopsis* for copper tolerance experiments.

The harm that heavy metals cause to plants is mainly derived through three modes of action. One is to replace protein ions and destroy the function of plant tissues. The second is its reaction with plant proteins to reduce protein activity. The third is to promote the production of ROS in cells and destroy the plant antioxidant system [71]. Our research was also carried out from these three aspects. First, we analyzed the expression of *BcGR1.1* under copper stress and found that the expression of *BcGR1.1* was significantly upregulated (Figure 3), which indicated that *BcGR1.1* was involved in the response to copper stress. A copper stress tolerance test was carried out with stable transgenic Arabidopsis lines: OE3, OE7 and OE8. It was found that: 1. *BcGR1.1* transgenic lines had less damage to the root system than WT, showing higher biomass and longer root length (Figure 5b,c). 2. Under copper stress, the protein activities of *BcGR1.1* transgenic lines were higher than that of WT, maintaining a higher level of GR and POD enzyme activities (Figure 5d and Figure 7b). 3. The ROS content of the *BcGR1.1* transgenic line under copper stress was lower than that of WT (Figure 7d). Combining the above three aspects, the heterologous overexpression of *BcGR1.1* in Arabidopsis was shown to increase the copper tolerance of Arabidopsis.

In addition, the ASA-GSH cycle involving GR is closely related to the plants response to adverse environments. Therefore, we tested the content and status of glutathione and AsA, and the results showed that, under copper stress, GSH/GSSG level was upregulated (Figure 6a) and AsA/DHA level was downregulated (Figure 6b) in the transgenic plants. These results indicated that, under copper treatment, the increase in GR activity allows plants to maintain high levels of GSH. At the same time, the AsA-GSH cycle was also affected, and more AsA was used to scavenge ROS, showing high levels of DHA content. These all improve the copper tolerance of plants.

## 5. Conclusions

In summary, the four homologous genes encoding GR in non-heading Chinese cabbage were divided into two types of localization. The cytoplasmic localization type *BcGR1.1* was the most responsive to copper stress. Roots were less damaged and the plant grew well in *BcGR1.1*-overexpressing *Arabidopsis*. Overall, *BcGR1.1* could regulate the AsA-GSH cycle, improve GSH accumulation, ASA utilization and some antioxidant enzyme activities, and then scavenge ROS caused by copper stress, thereby increasing the copper stress tolerance. The elucidation of this mechanism can provide a reference for breeders to select copper-tolerant crops, and make it possible to reuse soils with excessive copper content caused by heavy metals.

## Data Availability

Data is contained within the article and supplementary material.

## Supplementary Materials

The following supporting information can be downloaded at: https://www.mdpi.com/article/10.3390/antiox11020389/s1, Table S1: Primer sequence, Figure S1: Analysis of homologous gene expression in *BcGR1.1* silenced plant, Figure S2: Growth status of seedlings in the copper stress treatment pre-test, Figure S3: Tissue specific expression analysis of *BcGR1.1* (GUS staining).
