# Supplementary material for: BcGR1.1, a Cytoplasmic Localized Glutathione Reductase, Enhanced Tolerance to Copper Stress in Arabidopsis thaliana
Source: Antioxidants (Basel). 2022 Feb 15;11(2):389. doi: 10.3390/antiox11020389 (PMC8869148; doi:10.3390/antiox11020389)
Supplement: Supplementary file 1 [file antioxidants-11-00389-s001.zip › antioxidants-1565547-supplementary.pdf]

Supplementary material

Table S1. Primer sequence

Primer use	Primer name	Primer sequence
Gene cloning	BcGR1.1-F	ATGGGGAGGAAGATGCTTGCT
	BcGR1.1-R	TCATAGACTCGTCTTGGGTT
	BcGR1.2-F	ATGGCGAGGAAGATGCTTTCT
	BcGR1.2-R	TCATAGACTCGTTTGAGGTT
	BcGR2.1-F	ATGGCTACGACTCCGAAGCTG
	BcGR2.1-R	TTATTGCTCGGACTCCTCCTT
	BcGR2.2-F	ATGGCTTCGACTCCGAAGCTA
	BcGR2.2-R	CTAGACAGCTGTTTTAGCCTC
Promoter Cloning	ProBcGR1.1-F	AGATTTGCGATCGGATCAGAACCGT
	ProBcGR1.1-R	CGTCAGCAAGCATCTTCCTCCCCAT
Construction of subcellular localization vector	PR101-BcGR1.1-F	AAGTTCTTCACTGTTGATACATATG ATGGGGAGGAAGATGCTTGCT
	PR101-BcGR1.1-R	CCTCGCCCTTGCTCACCATGGATCC TAGACTCGTCTTGGGTT
	PR101-BcGR1.2-F	AAGTTCTTCACTGTTGATACATATG ATGGCGAGGAAGATGCTTTCT
	PR101-BcGR1.2-R	CCTCGCCCTTGCTCACCATGGATCC TAGACTCGTTTGAGGTT
	PR101-BcGR2.1-F	AAGTTCTTCACTGTTGATACATATG ATGGCTACGACTCCGAAGCTG
	PR101-BcGR2.1-R	CCTCGCCCTTGCTCACCATGGATCC TTGCTCGGACTCCTCCTT
	PR101-BcGR2.2-F	AAGTTCTTCACTGTTGATACATATG ATGGCTTCGACTCCGAAGCTA
	PR101-BcGR2.2-R	CCTCGCCCTTGCTCACCATGGATCC GACAGCTGTTTTAGCCTC
Construction of overexpression vector	PTCK303-BcGR1.1-F	CCGGATCCATGGGGAGGAAGATGCTTGCT
	PTCK303-BcGR1.1-R	CC GAGCTC TCATAGACTCGTCTTGGGTT
qPCR	qAtActin-F	TTGACAATTGATGCAAACAATGACG
	qAtActin-R	CCATTGCTTAATTCCACGGACAAAC
	qBcGAPC-F	AGAGCCGCTTCCTTCAACATCATT
	qBcGAPC-R	TGGGCACACGGAAGGACATACC

	qBcGR1.1-F	AAATGAGGGCACTTGTGGCT
	qBcGR1.1-R	AAATAGCACCACATCCGCCA
	qBcGR1.2-F	GAGAGCTTAACAAGGCCGCA
	qBcGR1.2-R	CAGAAAACCTAGCCGCACGA
	qBcGR2.1-F	CTCCATCTCTTCAAACCCTCTGC
	qBcGR2.1-R	AGTAAGGTGAGACGGGGACG
	qBcGR2.2-F	TTCGACTCCGAAGCTAACCAC
	qBcGR2.2-R	GGAGGGAACGAGAGGTTGGA

Hygromycin resistance gene detection primers	Hyg-F	GGTCGCGGAGGCTATGGATGC
	Hyg-R	GCTTCTGCGGGCGATTTGTGT

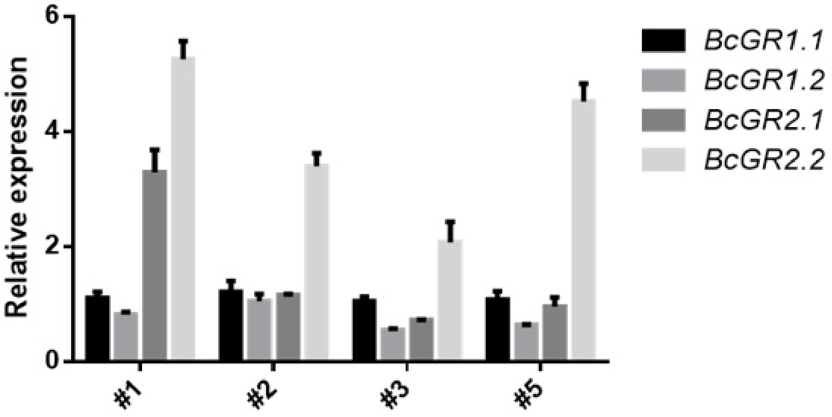

Figure S1. Analysis of homologous gene expression in *BcGR1.1* silenced plant.

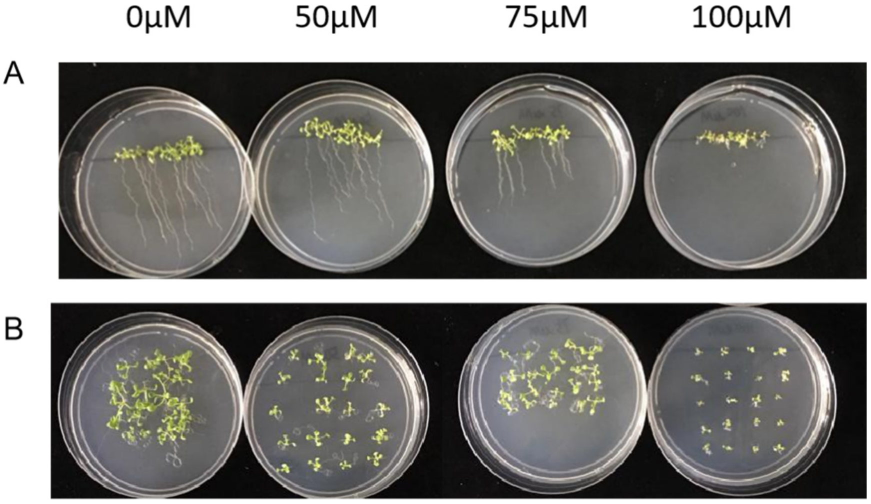

Figure S2. Growth status of seedlings in the copper stress treatment pre-test.

After sterilization, WT Arabidopsis seeds were evenly spread on 1/2MS, placed at 4 degrees for 2

days, and grown under normal growth conditions for 3 days. When the seeds have just germinated, placed them neatly on 1/2MS containing varying concentrations of copper as needed. WT of *Arabidopsis thaliana* was treated with different concentrations of copper and photographed after growing for 15 days. (A) : grow vertically after treatment; (B) : grow horizontally after treatment. Petri dishes are 9cm in diameter.

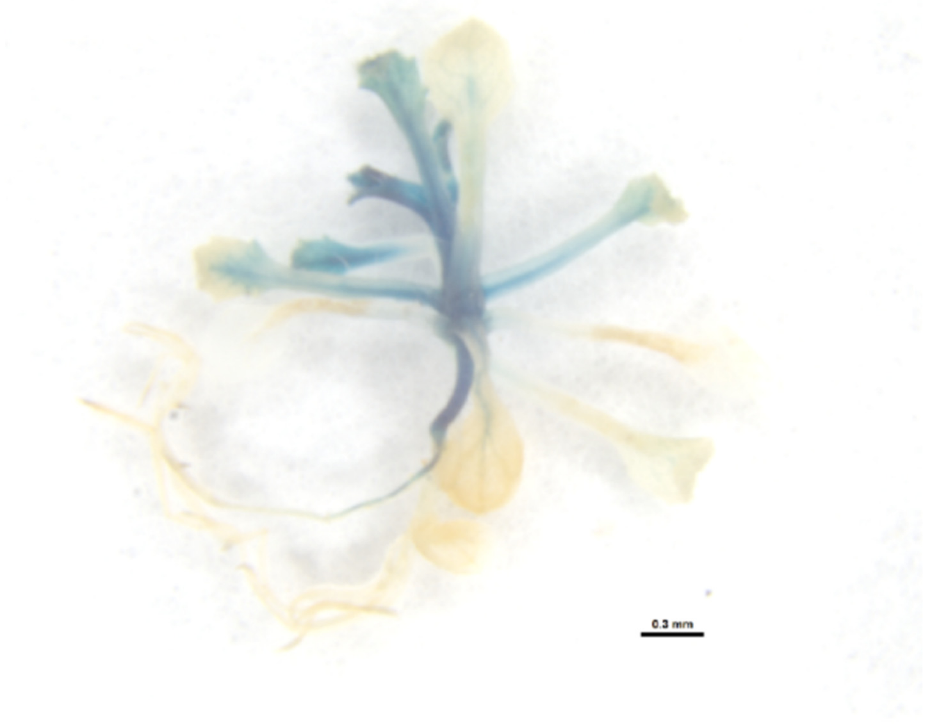

(b)

Figure S3. Tissue specific expression analysis of *BcGR1.1* (GUS staining).
We cloned the promoter sequence of *BcGR1.1*, constructed it in upstream of GUS, and transferred it into *Arabidopsis thaliana*. After screening, the positive seedlings were detected by GUS. Results in Figure 3b showed *BcGR1.1* is expressed in all tissues and organs. The highest expression was found in the stem, followed by the main root, young leaves and veins, and the lowest expressions were in old leaves and root hairs.
